# Oleanolic acid attenuates renal fibrosis in mice with unilateral ureteral obstruction via facilitating nuclear translocation of Nrf2

**DOI:** 10.1186/1743-7075-11-2

**Published:** 2014-01-06

**Authors:** Sungjin Chung, Hye Eun Yoon, Soo Jeong Kim, Sung Jun Kim, Eun Sil Koh, Yu Ah Hong, Cheol Whee Park, Yoon Sik Chang, Seok Joon Shin

**Affiliations:** 1 Department of Internal Medicine, College of Medicine, The Catholic University of Korea, 222 Banpo-daero, Seoul 137-701, Republic of Korea; 2 Division of Nephrology, The Catholic University of Korea Yeouido St. Mary’s Hospital, 10, 63-ro, Yeongdeungpo-gu, Seoul 150-713, Republic of Korea; 3 Division of Nephrology, The Catholic University of Korea Incheon St. Mary’s Hospital, 56, Dongsu-ro, Bupyeong-gu, Incheon 403-720, Republic of Korea; 4 Division of Nephrology, The Catholic University of Korea Seoul St. Mary’s Hospital, 222, Banpo-daero, Seoul 137-701, Republic of Korea; 5 Division of Nephrology, Korea University Guro Hospital, 148, Gurodong-ro, Guro-gu, Seoul 152-703, Republic of Korea

## Abstract

**Background:**

Renal interstitial fibrosis is a common final pathological process in the progression of kidney disease. This is primarily due to oxidative stress, which contributes to renal inflammation and fibrosis. Nuclear factor-erythroid-2-related factor 2 (Nrf2) is known to coordinate induction of genes that encode antioxidant enzymes. We investigated the effects of oleanolic acid, a known Nrf2 activator, on oxidative stress-induced renal inflammation and fibrosis.

**Methods:**

One day before unilateral ureteral obstruction (UUO) performed in C57BL/6 mice, oleanolic acid treatment was initiated and was continued until 3 and 7 days after UUO. Renal inflammation and fibrosis, markers of oxidative stress, and changes in Nrf2 expression were subsequently evaluated.

**Results:**

In the obstructed kidneys of UUO mice, oleanolic acid significantly attenuated UUO-induced collagen deposition and fibrosis on day 7. Additionally, significantly less inflammatory cell infiltration, a lower ratio of Bax to Bcl-2 expression, and fewer apoptotic cells on TUNEL staining were observed in the obstructed kidneys of oleanolic acid-treated mice. Oleanolic acid increased the expression of nuclear Nrf2, heme oxygenase-1, NAD(P)H:quinone oxidoreductase 1 and heat shock protein 70, and decreased lipid peroxidation in the obstructed kidney of UUO mice. There were no changes in the expression of total Nrf2 and Kelch-like ECH-associated protein 1, indicating that oleanolic acid enhanced nuclear translocation of Nrf2.

**Conclusions:**

These results suggest that oleanolic acid may exert beneficial effects on renal fibrosis by increasing nuclear translocation of Nrf2 and subsequently reducing renal oxidative stress.

## Background

Tubulointerstitial fibrosis is the final common pathological feature in the progression to end-stage renal disease irrespective of the type of primary glomerular injury, such as hypertensive nephrosclerosis, diabetic nephropathy or glomerulonephritis [1,2]. Over the last few years, numerous studies have been performed to identify the pathogenesis of renal fibrosis, and there has been increasing evidence that oxidative stress is an important factor in its progression. Reactive oxygen species (ROS) are produced in response to various insults to the kidney, and there are several reports that oxidative stress plays a significant role in renal damage in obstructed kidneys [3-5]. ROS cause tubulointerstitial injury by lipid peroxidation, increasing hydrogen peroxides, DNA breakdown, and protein damage [6]. ROS has also been found to play an important role in kidney fibrosis by regulation of inflammatory monocyte and macrophage infiltration, proliferation of interstitial fibroblasts, and extracellular matrix accumulation in the renal interstitium [6,7].

Oleanolic acid is a natural triterpenoid which has recently attracted considerable attention for its antioxidant properties. These properties have been tested in experimental models of drug-induced hepatotoxicity, multiple sclerosis, hypertension, atherosclerosis, and lung injury [8-14]. According to previous studies, the induction of nuclear factor-erythroid-2-related factor 2 (Nrf2) activation may mediate this oleanolic acid-induced antioxidant effect [8,15]. Nrf2 is a transcription factor that is widely known to be protective against oxidative stress and damage [16].

To gain further insight into the mechanisms that modulate renal fibrosis, we investigated whether up-regulation of Nrf2-dependent antioxidative signaling ameliorates renal inflammation and fibrosis. We used oleanolic acid, which is an Nrf2 activator, as the antioxidant in a mouse model of renal fibrosis induced by unilateral ureteral obstruction (UUO).

## Methods

### Animal preparation

All animal studies were conducted with the approval of The Institutional Animal Care and Use Committee seven- or eight-week-old male C57BL/6 mice weighting 20–25 g (OrientBio, Inc., Seoul, Republic of Korea) were used in this study. Animals were housed in standard cages in a room with constant temperature on a 12-hour light–dark cycle. They were fed a standard pellet laboratory chow (OrientBio, Inc., Seoul, Republic of Korea) and had free access to water. Oleanolic acid was dissolved in 2% w/v dimethyl sulfoxide (DMSO) and then diluted with distilled water for each injection to a final DMSO concentration of 0.2% w/v. Therefore, the vehicle used was 0.2% w/v DMSO. The dose of oleanolic acid was chosen based on a previous report [10]. Oleanolic acid or vehicle was administered intraperitoneally one day before UUO, and was continued for 3 or 7 days after surgery. UUO or sham surgery was performed as described previously [17]. In the UUO group, the left ureter was exposed through a mid-abdominal incision and ligated using 4–0 silk under general anesthesia. Sham-operated mice had their ureters exposed and manipulated without ligation. Mice were divided into a total of five groups as follows: sham, UUO-control day 3, UUO-oleanolic acid (OA) day 3, UUO-control day 7 and UUO-OA day 7 (n = 8, each group). Groups of mice were sacrificed at 3 and 7 days after the operation, and the obstructed kidneys were removed for tissue analysis.

### Quantitative determination of tissue collagen content

The total collagen content in kidney tissue was measured by acid hydrolysis of the kidney tissue section as described previously [18]. Briefly, each kidney sample was weighed, hydrolysed in 6 N HCl for 18 hours at 110°C, and dried thoroughly at 75°C. Dried samples were solubilized in citric acid collagen buffer (0.23 mol/L citric acid, 0.88 mol/L sodium acetate trihydrate, 0.85 mol/L sodium hydroxide, and 1.2% acetic acid) and filtered through a 0.45 μm centrifugal filter unit (Ultrafree-MC, Millipore, Billerica, MA, USA). Samples were diluted in citric acid collagen buffer and then loaded into each well of a microplate, to which 100 μL of fresh chloramine-T solution (1.4% chloramine-T and 10% n-propanol in citric acid buffer) was added to start the oxidation reaction. The microplate was incubated for 15 min at room temperature, and 100 μL of Ehrlich’s reagent (15% 4-dimethylamino-benzaldehyde, 62% n-propanol, and 18% perchloric acid) was added to each well to start the colour reaction. The microplate was incubated in a large water bath for 20 min at 65°C. The amount of hydroxyproline was measured by a spectrophotometric assay at 550 nm. Total collagen in the kidney tissue was calculated on the assumption that collagen contains 12.7% hydroxyproline by weight.

### Histologic examination

Histologic analyses were performed using paraffin-embedded tissues. To evaluate the severity of tubular injury and tubulointerstitial fibrosis, kidney sections were processed and stained with hematoxylin and eosin (H&E) and Masson trichrome, respectively. Renal tubular injury was assessed and given a score from 0 to 3 (0 = normal, 1 = slight damage, 2 = moderate damage, 3 = severe damage) as previously described [19]. Fibrotic area was quantified using MetaMorph imaging software (Molecular Devices Inc., Downingtown, PA, USA) in more than 10 randomly selected fields from cortex and medulla. The ratio of the fibrotic area to the total selected field was indicated as the severity of tubulointerstitial fibrosis. Immunohistochemistry for F4/80 and TdT-mediated dUTP nick end labelling (TUNEL) were performed. After deparaffinization, the sections were hydrated, incubated in 0.5% Triton X-100–PBS solution for 30 minutes, and washed three times with PBS. Nonspecific binding sites were blocked with normal donkey serum diluted 1:10 in PBS for 1 hour, and the sections were incubated overnight in a humidified chamber at 4°C with primary antibody raised against F4/80 (rat anti-mouse F4/80 monoclonal antibody, Abcam, Cambridge, MA, USA). After rinsing in PBS, the sections were incubated in peroxidase-conjugated anti-mouse IgG (Jackson ImmunoResearch Laboratories, West Grove, PA, USA) for 1 hour. For coloration, the sections were incubated with a mixture of 0.05% 3,3′-diaminobenzidine containing 0.01% H_2_O_2_ at room temperature until a brown color was visible, and were then washed with Tris buffer (pH 7.6), counterstained with hematoxylin, and observed under light microscopy (Zeiss LSM 510, Carl Zeiss, Jena, Germany). Twenty high-power fields that included the renal cortex and corticomedullary junction were randomly selected in each section, and proportional areas of staining were quantified using MetaMorph imaging software (Molecular Devices Inc., Downingtown, PA, USA). Apoptotic cells were identified using a TUNEL assay. This was performed on paraffin sections fixed with 4% paraformaldehyde according to the manufacturer’s instructions (Millipore, Billerica, MA, USA). TUNEL-positive apoptotic cells were counted in 20 randomly selected tubulointerstitial areas of each kidney sample. All slides were analyzed in a blinded fashion.

### Western blot analysis

The total protein content of the renal tissues was extracted using a Pro-Prep Protein Extraction Kit (iNtRON Biotechnology, Inc., Seongnam, Gyeonggi-do, Republic of Korea) according to the manufacturer’s instructions. The protein concentration was determined using a BCA protein assay (Pierce Biotechnology, Rockford, IL, USA). Equal kidney protein samples were separated by 15% SDS-PAGE and transferred to nitrocellulose membranes. Nuclear extracts, which were used for Nrf2 immunoblotting, were also prepared with the NE-PER nuclear kit as previously described (Pierce Biotechnology, Rockford, IL) [8]. For immunodetection, the blots were incubated overnight in PBS containing 0.1% Tween-20 and 5% skim milk with primary antibodies raised against the following proteins: heme oxygenase (HO)-1 (diluted 1:200, BD Biosciences, San Jose, CA, USA), heat shock protein 70 (Hsp70) (1:1000, ENZO Life Sciences, Inc., Farmingdale, NY, USA), NAD(P)H:quinone oxidoreductase 1 (NQO1) (1:500, Santa Cruz Biotechnology, Santa Cruz, CA, USA), MnSOD (1:2000, Abcam, Cambridge, MA, USA), catalase (1:2000; Abcam), Bax (1:500, Santa Cruz Biotechnology), Bcl-2 (1:100, Santa Cruz Biotechnology), Kelch-like ECH-associated protein 1 (Keap1; 1:1000; Abcam), and Nrf2 (1:1000; Abcam). The blots were washed and incubated with a secondary antibody conjugated with horseradish peroxidase (Jackson ImmunoResearch Laboratories, West Grove, PA, USA), and the protein bands were visualized using the chemiluminescence detection system (ImageQuant LAS 4000 mini, GE Healthcare, Piscataway, NJ, USA). Intensity values were determined using Image-Pro Plus software (Media Cybernetics, Bethesda, MD, USA).

### Measurement of oxidative stress

To determine the level of lipid peroxidation in the kidney tissues, the level of malondialdehyde (MDA), a biomarker of free radical-mediated oxidative stress, was measured as previously described [20]. Briefly, the kidney tissue was homogenized in sucrose buffer (70 mM sucrose, 210 mM mannitol, 1 mM EDTA, 10 mM Hepes in distilled water). After protein quantification, 100 μg of kidney lysates were mixed with 1 ml of thiobarbituric acid-trichloroacetic acid-HCl solution (0.375% thiobarbituric acid, trichloroacetic acid in 0.25 N HCl, pH 2.0) and boiled at 100°C for 15 minutes. Absorbance was measured at a wavelength of 535 nm. The hydrogen peroxide (H_2_O_2_) levels in the kidney tissues were determined using Fox reagent (0.25 M H2SO4, 1 M sorbitol, 25 mM ferrous ammonium sulfate, and 1 mM xylenol orange in distilled water) as previously described [20]. H_2_O_2_ oxidizes iron (II) to iron (III) in the presence of sorbitol, which acts as a catalyst for the reaction. Iron (III) then forms a purple complex with xylenol orange. Absorbance was measured at a wavelength of 560 nm.

### Statistical analysis

Data are expressed as means ± SE. Statistical differences among groups were calculated using ANOVAs with Bonferroni correction. P-values of < 0.05 were considered to be statistically significant.

## Results

### Tubular injury, interstitial fibrosis, total collagen content and interstitial inflammatory cell infiltration in renal tissue

**Figure 1:**
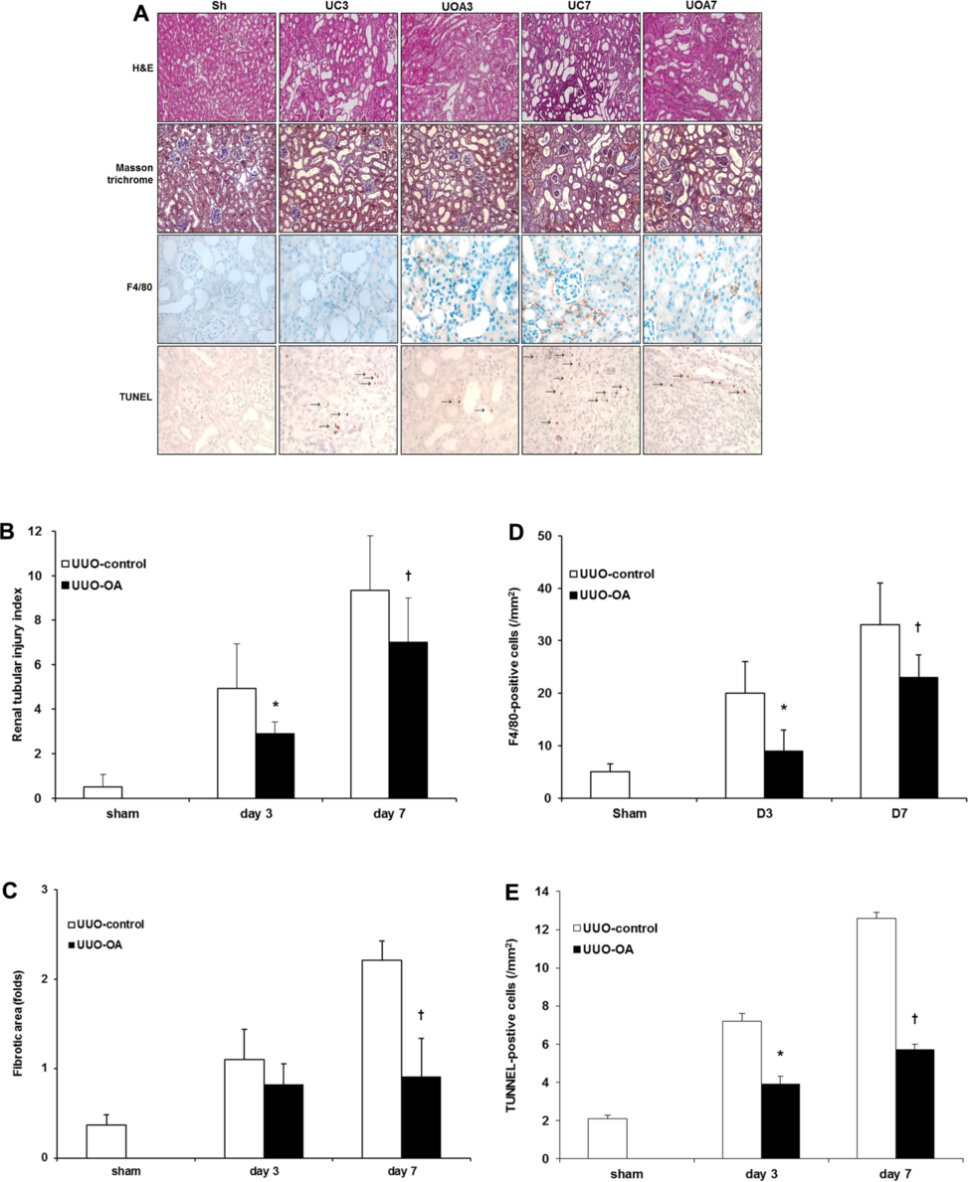
**Effects of oleanolic acid (OA) on renal morphological changes in unilateral ureteral obstruction (UUO). (A)** Representative photographs assessing renal tubular injury (H&E, x200), interstitial fibrosis (Masson trichrome, x200), immunohistochemical staining for the infiltration of F4/80-positive cells (x400) and TUNEL staining (x400). Black arrows indicate TUNEL-positive cells (brown color). **(B)** Renal tubular injury index. **(C)** Semiquantitative analysis of interstitial fibrosis. **(D)** Number of F4/80-positive cells. **(E)** Number of TUNEL-positive cells. Sh, sham; UC3, UUO-control day 3; UOA3, UUO-oleanolic acid day 3; UC7, UUO-control day 7; UOA7, UUO-oleanolic acid day 7; *P < 0.05 versus UUO-control day 3 group; ^**†**^*P* < 0.05 versus UUO-control day 7 group.

Tubular injury in the obstructed kidney was markedly increased after UUO (Figure 1A). Oleanolic acid significantly decreased tubular injury in the obstructed kidney on both day 3 and day 7 (Figure 1B). The severity of renal fibrosis also increased after UUO in a time-dependent manner, as seen in Masson trichrome staining (Figure 1A). The degree of tubulointerstitial fibrosis tended to be lower in the UUO-OA mice compared to those of UUO-control mice on day 3. However, this difference was not statistically significant. In contrast, the tubulointerstitial fibrotic areas in the obstructed kidneys from UUO-OA prominently decreased compared to those from UUO-control mice on day 7 (Figure 1C).

Immunohistochemistry for F4/80 was performed to investigate the degree of interstitial inflammatory cell infiltration. The number of F4/80-positive cells increased according to time course of UUO (Figure 1A). Oleanolic acid markedly decreased renal F4/80-positive cells infiltration in the obstructed kidneys from UUO-OA mice on both day 3 and day 7 (Figure 1D).

**Figure 2:**
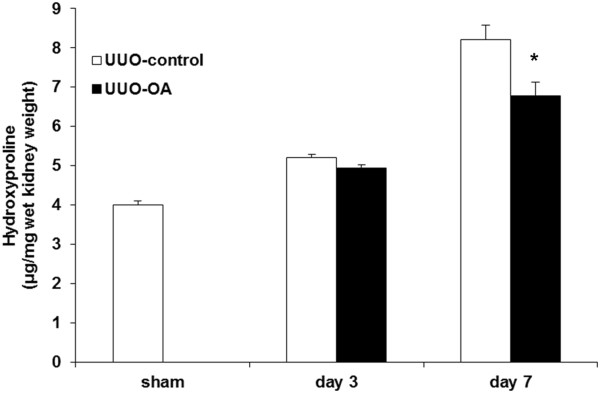
**Levels of hydroxyproline in the obstructed kidney after unilateral ureteral obstruction (UUO) with or without oleanolic acid (OA) treatment.** *P < 0.05 versus UUO-control day 7.

The amount of total collagen in the obstructed kidneys was found to be increased after UUO. Oleanolic acid significantly decreased the total collagen content in the obstructed kidneys from UUO-OA mice compared to that from UUO-control mice on day 7 (Figure 2).

### Keap1 and Nrf2 expressions

**Figure 3:**
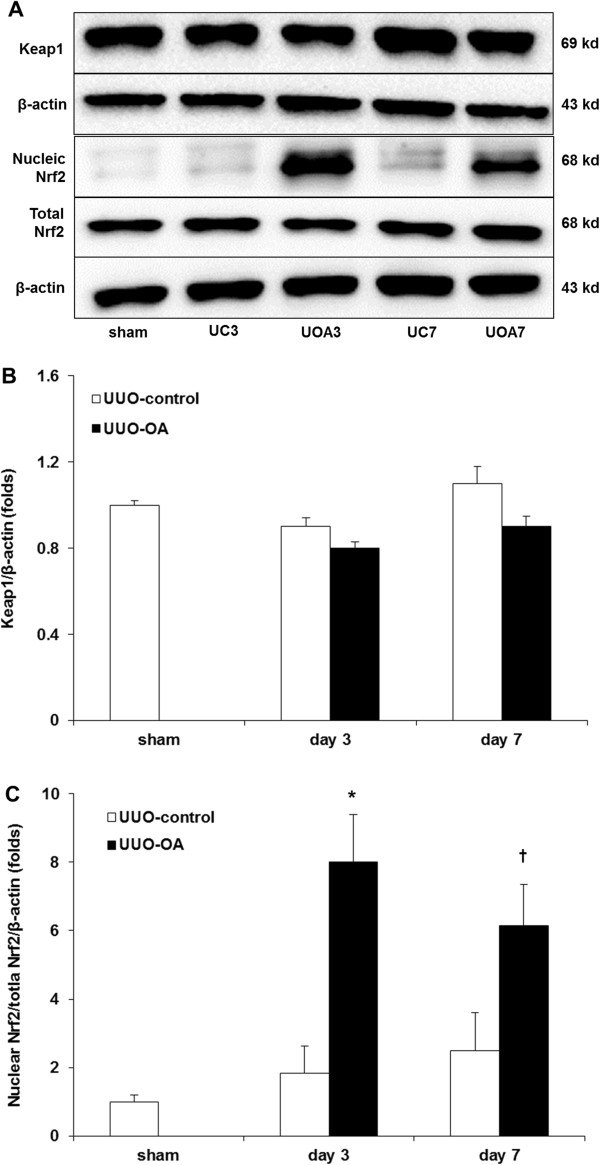
**Effects of oleanolic acid (OA) on Keap1 and Nrf2 expression in the obstructed kidney. (A)** Representative Western blots of Keap1, total Nrf2 and nuclear Nrf2 in the obstructed kidney after unilateral ureteral obstruction (UUO) with or without OA treatment. **(B)** The immunofold of the expression of Keap1. **(C)** The ratio of the expression of nuclear Nrf2 to total **Nrf2.** UC3, UUO-control day 3; UOA3, UUO-oleanolic acid day 3; UC7, UUO-control day 7; UOA7, UUO-oleanolic acid day 7; *P < 0.05 versus UUO-control day 3 group; ^**†**^*P* < 0.05 versus UUO-OA day 7 group.

The levels of intra-renal Keap1 and Nrf2 were determined using western blot analysis (Figure 3A). Under basal conditions, Nrf2 is located in the cytoplasm as an inactive complex bound to a repressor molecule, Keap1 [8]. Various stimuli including oxidants and antioxidants result in dissociation of Nrf2 from Keap1 and its translocation to the nucleus [16]. The expression of Keap1 was not changed after UUO, and oleanolic acid did not change the level of Keap1 in UUO mice on both day 3 and day 7 (Figure 3B). The expression of total Nrf2 showed a pattern similar to that of Keap1 (Figure 3A). On the contrary, oleanolic acid markedly increased the levels of nuclear Nrf2 in the obstructed kidneys. Therefore, nuclear Nrf2/total Nrf2 ratio was significantly increased in the UUO-OA mice compared with those of UUO-control mice on both days 3 and day 7 (Figure 3C).

### Renal oxidative stress

**Figure 4:**
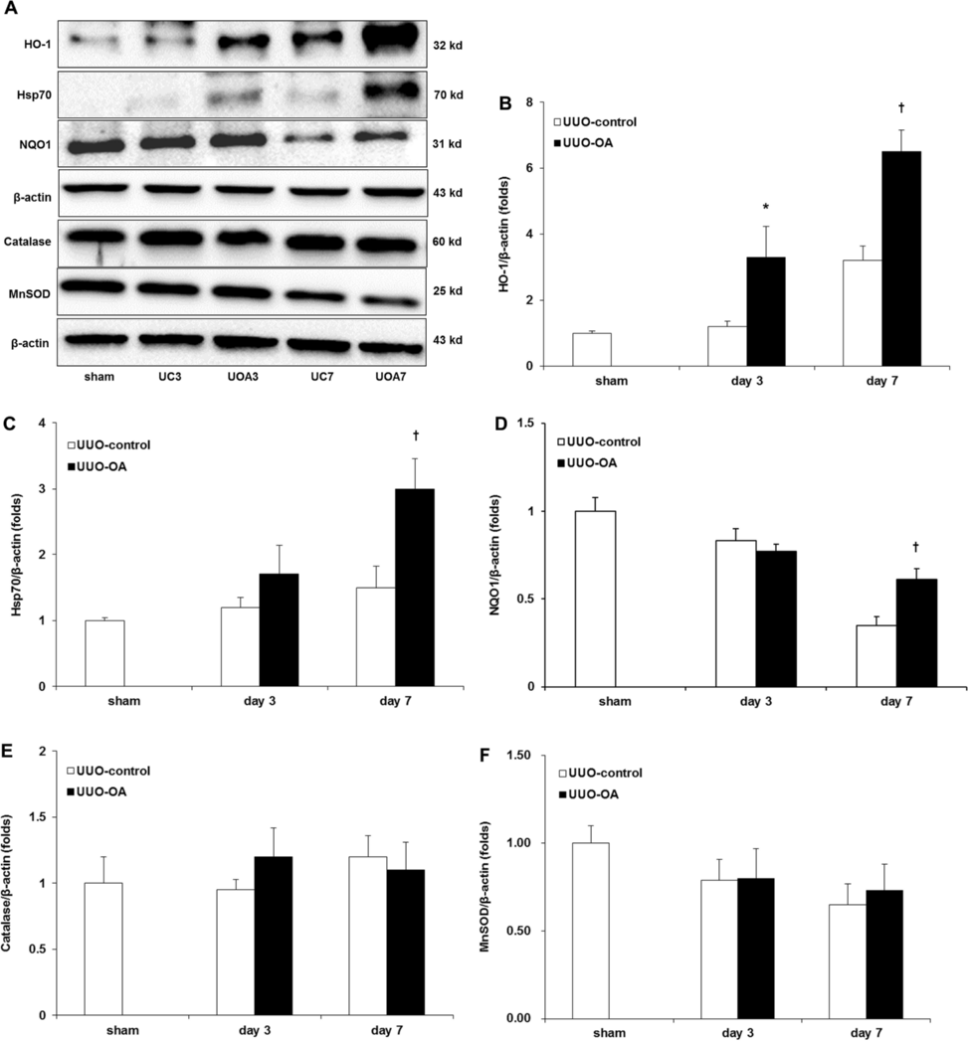
**Effects of oleanolic acid (OA) on HO-1, Hsp70, NQO1 and catalase expression in the obstructed kidney. (A)** Representative Western blots of HO-1, Hsp70, NQO1 and catalase in the obstructed kidney after unilateral ureteral obstruction (UUO) with or without OA treatment. **(B)** The immunofold of the expression of HO-1. **(C)** The immunofold of the expression of Hsp70. **(D)** The immunofold of the expression of NQO1. **(E)** The immunofold of the expression of catalase. **(F)** The immunofold of the expression of MnSOD. UC3, UUO-control day 3; UOA3, UUO-oleanolic acid day 3; UC7, UUO-control day 7; UOA7, UUO-oleanolic acid day 7. *P < 0.05 versus UUO-control day 3 group; ^**†**^*P* < 0.01 versus UUO-control day 7 group.

Oxidative stress induced by UUO was assessed by measuring the expression of HO-1, Hsp70, NQO1, catalase, and MnSOD using western blot analysis (Figure 4A), as well as the renal tissue levels of H_2_O_2_ and lipid peroxidation. The expression of HO-1 showed increased levels after UUO according to time dependent manner. It was significantly greater in the obstructed kidneys of UUO-OA mice compared to that of UUO-control mice on both day 3 and day 7 (Figure 4B). The expression of Hsp70 showed no significant differences between sham and UUO-control mice. Oleanolic acid did not significantly increase the expression of Hsp70 on day 3. However, it markedly increased the level of Hsp70 on day 7 (Figure 4C). The expression of NQO1 was decreased after UUO; however, oleanolic acid significantly increased the level of NQO1 on day 7 (Figure 4D). On the other hand, the expression of catalase, an H_2_O_2_-inducible anti-oxidant enzyme, was not different between groups, and oleanolic acid showed no effect on the levels of catalase (Figure 4E). The expressions of MnSOD in experimental groups showed similar patterns to those of catalase (Figure 4F).

**Figure 5:**
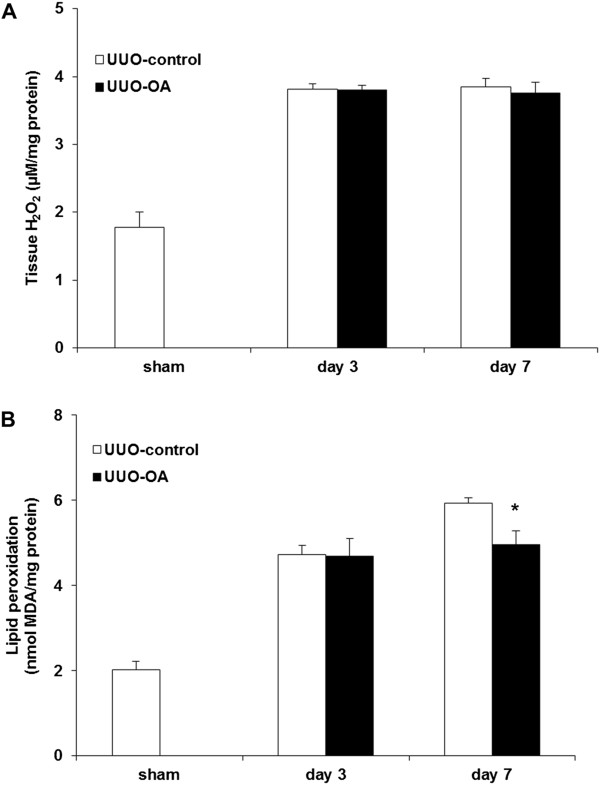
**Effect of oleanolic acid (OA) on H**_**2**_**O**_**2 **_**formation and lipid peroxidation in the obstructed kidney after unilateral ureteral obstruction (UUO). (A)** Levels of tissue H_2_O_2_. **(B)** Levels of lipid peroxidation. *P < 0.05 versus UUO-control day 7.

Direct measurement of the tissue levels of H_2_O_2_ and lipid peroxidation in the obstructed kidneys significantly increased after UUO. Similar to the intra-renal expression of catalase, the tissue levels of H_2_O_2_ were not different in the obstructed kidneys treated with or without OA on both day 3 and day 7 (Figure 5A). Oleanolic acid did not lower the renal tissue levels of lipid peroxidation in the UUO-OA mice on day 3. However, it significantly decreased the levels of lipid peroxidation in the obstructed kidneys from UUO-OA mice on day 7 (Figure 5B).

### Renal apoptosis

**Figure 6:**
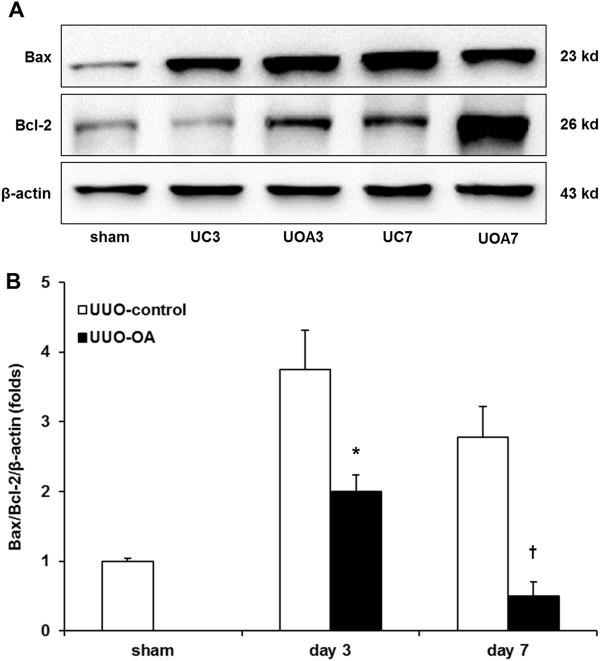
**The effects of oleanolic acid (OA) on Bax and Bcl-2 expression in the obstructed kidney. (A)** Representative Western blots of Bax and Bcl-2 in the obstructed kidney after unilateral ureteral obstruction (UUO) with or without OA treatment. **(B)** The ratio of the expression of Bax to Bcl-2. UC3, UUO-control day 3; UOA3, UUO-oleanolic acid day 3; UC7, UUO-control day 7; UOA7, UUO-oleanolic acid day 7. *P < 0.01 versus UUO-control day 3 group; ^**†**^*P* < 0.05 versus UUO-control day 7 group.

To investigate whether the nuclear translocation of Nrf2 and the activation of antioxidant enzyme have effects on renal apoptosis, we examined the expression of the pro-apoptotic protein Bax and the anti-apoptotic protein Bcl-2 by western blot analysis (Figure 6A), together with renal TUNEL-positive cells. UUO increased the Bax expression according to the time course of renal damage. However, there was no significant difference in the renal Bax expression between UUO-control mice and UUO-OA mice. The Bcl-2 expressions were slightly increased after UUO in a time dependent manner. These increases were more prominent after OA treatment on both day 3 and day 7 (Figure 6B).

The number of TUNEL-positive cells increased according to time course of UUO. Oleanolic acid markedly decreased renal TUNEL-positive cells in the obstructed kidneys from UUO-OA mice on both day 3 and day 7 (Figure 1A and 1E).

## Discussion

In the present study, treatment with oleanolic acid attenuated renal inflammation and fibrosis in UUO mice. This study also demonstrated that oleanolic acid suppressed oxidative stress and cellular apoptosis, and promoted antioxidant enzyme expression which may be induced by enhancement of nuclear translocation of cytosolic Nrf2.

Oxidative stress plays a significant role in the progression of tubulointerstitial damage in UUO-induced renal injury [21]. Chronic kidney disease characterized by fibrosis is related to decreased expression of superoxide dismutase and increased expression of NADPH oxidase [22]. UUO also increased lipid peroxidation, shown as the levels of MDA, in the obstructed kidneys, and pretreatment with a superoxide dismutase mimetic reduced the increase in tissue lipid peroxidation after UUO [6]. Excess ROS production from damaged tubular cells or infiltrated leukocytes such as ED-1-positive macrophages leads to renal tubular apoptosis in kidneys after UUO [23]. In the present study, oleanolic acid significantly reduced renal tissue lipid peroxidation and tubular apoptosis, suggesting that oleanolic acid would play a preventative role in the oxidative stress pathway induced by UUO.

Oleanolic acid is a natural triterpenoid that is a constituent of the leaves of *Olea europaea, Viscum album L.* and other plants [8], and has been used as a traditional medicine [24]. It is also found in promace olive oil and is the principal source of fat in the Mediterranean diet, supporting the hypothesis that some components of olive oil may have beneficial effects on human health [9]. Recently, several studies have shown the therapeutic properties of oleanolic acid. One study demonstrated an inhibitory effect of oleanolic acid on the production of advanced glycation end-products in the kidneys of streptozotocin-induced diabetic mice [25]. A previous study reported that oleanolic acid improved neurological symptoms in mice with experimental autoimmune encephalomyelitis [9]. The effects of oleanolic acid are thought to result from its anti-inflammatory or anti-oxidant activity [26]. For example, a recent study reported that treatment with oleanolic acid had beneficial effects on the development of atherosclerosis in apolipoprotein E knockout mice, which may be due to its antioxidant properties [11]. Another study demonstrated that oleanolic acid treatment reduced blood pressure in hypertensive animals via endothelium-dependent vasodilatation mediated by nitric oxide [10]. In our study, oleanolic acid reduced total collagen accumulation and interstitial macrophage infiltration in the obstructed kidneys. It also attenuated UUO-induced renal oxidative stress, possibly by up-regulating HO-1, NQO1 and Hsp70 expression and reducing lipid peroxidation. This suggests that oleanolic acid alleviates renal inflammation and fibrosis by decreasing oxidative stress in mice with UUO.

Activation of Nrf2 has been shown to be responsible for oleanolic acid-mediated protection against various insults [8,15]. In a recent study, the synthetic triterpenoid analog of oleanolic acid, CDDO-Im, was used in mice fed a high-fat diet. Treatment with CDDO-Im prevented high-fat diet-induced obesity in wild-type mice, but not in Nrf2-disrupted mice [15]. This finding suggests that oleanolic acid targets Nrf2 signaling. Keap1, a cysteine-rich protein, plays an important role in the inhibition of nuclear translocation of Nrf2 as well as facilitating proteasomal degradation of Nrf2 [9]. Under normal conditions, Nrf2 is sequestered in the cytoplasm via binding to its repressor molecule, Keap1 [16]. Stressful conditions such as oxidative or ER stress induce the dissociation of the Nrf2-Keap1 complex, and subsequent nuclear translocation of Nrf2. This is thought to be the mechanism of Nrf2 activation. An earlier study reported that oleanolic acid facilitated Nrf2 nuclear accumulation, which contributed to protection from acetaminophen hepatotoxicity [8]. Compatible with the study, our results showed that oleanolic acid enhanced the expression of nuclear Nrf2, resulting in an increased nuclear Nrf2/total Nrf2 ratio in the obstructed kidneys. In the present study, UUO or oleanolic acid treatment did not change the renal expression of Keap1 and total Nrf2 in the obstructed kidneys. On the other hand, a previous study demonstrated a paradoxical reduction of inactivated Nrf2, accompanied by a significant elevation of Keap1 in cases of severe oxidative stress or inflammation [16]. Although the reason for this discrepancy remains unclear, the changes in Keap1 and total Nrf2 levels may differ according to experimental models of oxidative stress and the severity of renal injury. Taken together, our results may suggest that oleanolic acid facilitates nuclear translocation of cytosolic Nrf2 rather than directly inducing dissociation of the Nrf2-Keap1 complex.

Within the nucleus, Nrf2 binds to regulatory sequences called antioxidant response elements or electrophile response elements, which are located in the promoter region of genes encoding the antioxidant and phase 2 detoxifying enzymes such as glutathione-S-transferases, NQO1, glutamate-cysteine ligase catalytic subunit, catalase, thioredoxin, and HO-1 [8,16]. The present study showed that oleanolic acid markedly increased the expression of HO-1, an important antioxidant enzyme, in the obstructed kidneys of oleanolic acid-treated mice. In general, HO-1 metabolizes heme that accumulates in tissues as a by-product of red blood cell turnover. Metabolites produced by such degradation reactions have been known to trigger signaling cascades that aid antioxidant defenses and protect against pathological increases in oxidative stress [27]. Unlike HO-1, in our results, expression of NQO1 upregulated by oleanolic acid was more prominent on day 7 than on day 3. There appears to be some differences in the expression of phase 2 enzymes according to the experimental model: the regulatory mechanisms of phase 2 enzymes in relation to the Nrf2 pathway may be tissue- or cell-specific [28]. Our results suggest that HO-1, rather than NQO1, plays a major role in the attenuation of renal inflammation and fibrosis in this UUO model. We also observed that oleanolic acid treatment increased Hsp70 protein expression in renal tissue on UUO day 7. This finding accords with previous results that activation of Nrf2 upregulates its downstream gene products and the Hsp70 gene [29]. Our results demonstrated that oleanolic acid improved interstitial fibrosis and the total collagen content of renal tissue on day 7 after UUO, although attenuation of tubular injury and interstitial inflammation by oleanolic acid was evident even in day 3. Considering that renal fibrosis in our study was further attenuated on day 7 than it was on day 3, late upregulation of NQO1 and Hsp70 might have synergistic effects on the suppression of progressive fibrosis resulting from UUO. On the other hand, there was no difference in the expression of catalase, which is responsible for H_2_O_2_ neutralization, in obstructed kidneys with or without OA treatment. It is unclear why oleanolic acid did not change the expression of catalase together with the levels of tissue H_2_O_2_ in this study. In addition, MnSOD protein level showed no differences with administration of oleanolic acid. Taking findings of previous studies into consideration [6,12], we cautiously speculate that the type of oxidative stress may influence the detailed action of Nrf2 that regulates induction of genes encoding specific antioxidants.

The present study demonstrated that renal apoptosis was attenuated by treatment with oleanolic acid. This finding is consistent with a recent study showed that the up-regulation of HO-1 significantly decreased cellular apoptosis [30]. Our study also showed that although oleanolic acid had no effect on the levels of the pro-apoptotic enzyme Bax it increased the expression of the anti-apoptotic enzyme Bcl-2, resulting in a decreased Bax/Bcl-2 ratio. It has been suggested that Nrf2 targets the anti-apoptotic Bcl-2 protein, and that antioxidant treatment releases Nrf2 and increases Bcl-xL heterodimerization with Bax, resulting in reduced cellular apoptosis [31]. Our results suggest that HO-1 and Bcl-2 may serve as key players in Nrf2-upregulated cell survival under oxidative stress induced by UUO. Furthermore, the interaction between Hsp70 and Bcl-2 is considered to contribute to renoprotection in this study. It has been known that Hsp70 can inhibit apoptosis by antagonizing the apoptosis-inducing factor [32].

The current study has some limitations. First, we did not measure the blood urea nitrogen (BUN) and serum creatinine as renal functional parameters. However, many previous studies have reported that BUN or serum creatinine was not significantly affected by UUO because of the presence of a contralateral kidney with good renal function [33,34], suggesting that BUN and serum creatinine are not good indicators of renal function in an animal model of UUO. Second, we could not evaluate the molecular mechanism through which oleanolic acid modulates nuclear translocation of Nrf2. Third, we did not investigate the possible Nrf2-independent mechanisms that could contribute to renoprotection in the UUO model. Like a previous report [8], we found that oleanolic acid treatment increased the expression of Hsp70 protein that may contribute to Nrf2-independent renoprotection. A previous study used microarray to compare the overall gene expression signatures modulated by pharmacologic or genetic activation of Nrf2 signaling in liver tissue and found several Keap1-Nrf2-independent genes [35]. Another report indicated that oleanolic acid protected against hepatotoxicity in wild type mice, but did so to a lesser degree in Nrf2-null mice, suggesting that oleanolic acid could activate Nrf2-independent protective mechanisms [8]. Further investigation is needed to elucidate the exact mechanisms of the Nrf2-independent effects of oleanolic acid.

## Conclusions

In conclusion, we found that oleanolic acid treatment protects against oxidative insults in obstructed kidneys after UUO. Oleanolic acid appears to exert its beneficial effects by facilitating nuclear translocation of Nrf2 and subsequently up-regulating antioxidant and anti-apoptotic enzymes. Treatment with oleanolic acid may provide a therapeutic approach in preventing renal oxidative stress, inflammation, and fibrosis.

## Competing interests

The authors declare that there are no competing interests.

## Authors’ contributions

The experiment was designed and implemented by SC and SJS. Data were analyzed by HEY, SJK, SJK, ESK, YAH, CWP, YSC and SJS. SC and SJS prepared the manuscript. SC and SJS supervised overall project. All authors read and approved the final version of manuscript.
